# Multi‐omics reveals different signatures of obesity‐prone and obesity‐resistant mice

**DOI:** 10.1002/imo2.59

**Published:** 2025-02-04

**Authors:** Congcong Wang, Jinhua Lin, Meng Duan, Jialing He, Simayi Halizere, Ningxin Chen, Xinyu Chen, Ye Jiao, Wei He, Kenneth A. Dyar, Fei Yang, Shankuan Zhu

**Affiliations:** ^1^ Department of Nutrition and Food Hygiene, Children's Hospital Zhejiang University School of Medicine, National Clinical Research Center for Child Health Hangzhou China; ^2^ Chronic Disease Research Institute, School of Public Health, School of Medicine Zhejiang University Hangzhou China; ^3^ Metabolic Physiology, Institute for Diabetes and Cancer, Helmholtz Diabetes Center, Helmholtz Zentrum München, German Research Center for Environmental Health Neuherberg Germany; ^4^ German Center for Diabetes Research (DZD) Neuherberg Germany; ^5^ School of Medicine and Health Technical University of Munich Munich Germany; ^6^ Binjiang Institute of Zhejiang University Hangzhou China

**Keywords:** high‐fat diet, metabolomics, microbiota, obesity, transcriptome

## Abstract

Obesity‐prone (OP) and obesity‐resistant (OR) individuals demonstrate significant metabolic differences, potentially influenced by variations in the gut microbiome. However, the influence of host–microbiota interactions on obesity susceptibility remains unknown. We performed an integrative multi‐omics approach to explore microbial, metabolic, and genetic differences in high‐fat diet (HFD)‐fed OP and OR mice, with additional analyses of gut microbiota variations in humans. In OP mice, the dynamic gut microbiota was characterized by a stable presence of *Longibaculum*, while *Kineothrix* predominated in OR mice. We termed both as keystone bacteria. Beyond these, eight dominant bacterial genera were significantly associated with bile acid metabolites and amino acids. Three of these genera were also identified in OR humans and showed positive correlations with genes that may support intestinal barrier function. We identified 22 specific amino acid profiles as potential biomarkers for obesity susceptibility, along with significantly increased levels of ten non‐12‐OH bile acids in fecal of OR mice. In vivo, mouse experiments demonstrated that ursodeoxycholic and hyodeoxycholic acids could reduce HFD‐induced obesity. Additionally, the colon of OP mice displayed a higher presence of inflammatory cells. These findings suggest that host–microbiota interactions may contribute to phenotypic differences between OP and OR. Our study offers insights into crucial intestinal markers associated with obesity, providing a valuable resource for advancing the understanding of obesity‐prone and obesity‐resistant phenotypes.

## Highlights

- Gut microbiota dynamics were described in obesity‐prone (OP) and obesity‐resistant (OR) mice, with *Longibaculum* and *Kineothrix* identified as keystone bacteria.
- *Kineothrix*, *Intestinimonas*, and *Fournierella*, which observed in OR mice, were also detected in OR humans.
- Ten non‐12‐OH bile acids, including ursodeoxycholic and hyodeoxycholic acids, were elevated in OR mice and reduced weight gain induced by a high‐fat diet.
- Twenty‐two specific amino acid profiles were identified as potential biomarkers for obesity susceptibility.

## Graphical abstract

This study explores gut microbiota dynamics and metabolic signatures in obesity‐prone (OP) and obesity‐resistant (OR) mice. *Longibaculum* and *Kineothrix* were identified as keystone bacteria in the gut microbiota dynamics of OP and OR mice, respectively. Notably, bacteria such as *Kineothrix* and *Intestinimonas* observed in OR mice were also detected in obesity‐resistant humans, suggesting cross‐species relevance. Metabolomic analysis revealed that ten non‐12‐OH bile acids, including ursodeoxycholic acid (UDCA), were elevated in OR mice, contributing to weight regulation. Additionally, 22 specific amino acid profiles differentiated OP and OR mice, highlighting potential biomarkers for obesity susceptibility. Integrated analyses of the microbiome, metabolome, and colon transcriptome revealed a dynamic interplay among gut microbiota, bile acids, amino acid metabolism, and gut barrier function, providing both theoretical insights and data support for understanding obesity resistance mechanisms.

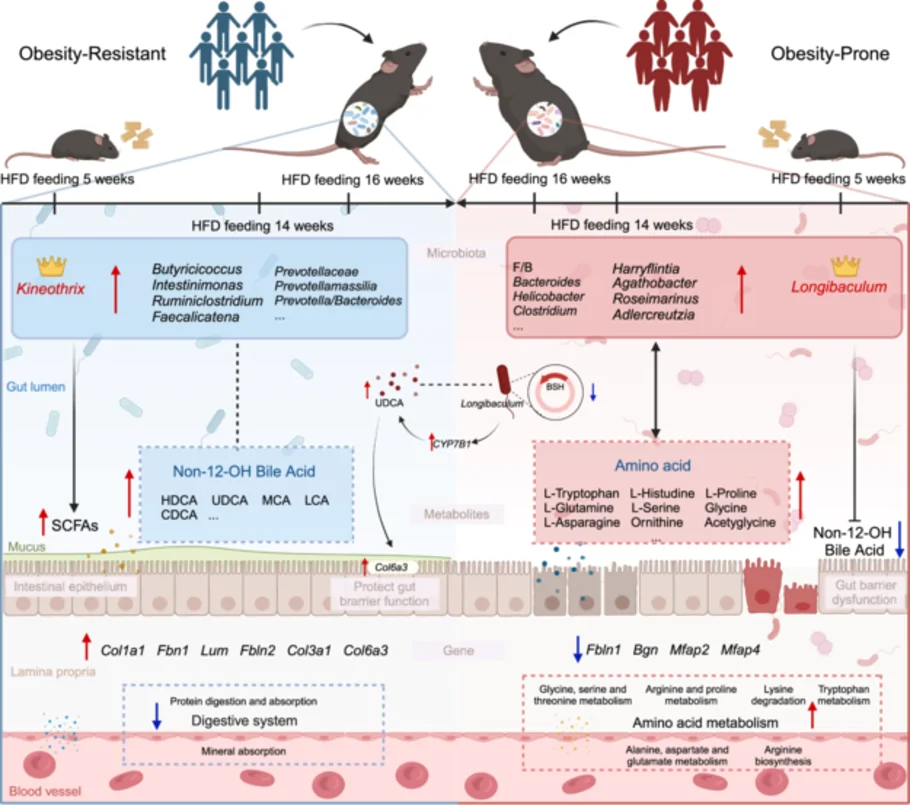

## 1 INTRODUCTION

Obesity is a widespread global health issue and serves as a significant risk factor for type 2 diabetes, cardiovascular diseases, and certain types of cancer. Beyond genetic regulation, environmental factors, particularly the overconsumption of high‐calorie food diets, contribute substantially to the progression of obesity [1]. Chang et al. discovered that despite being fed an identical high‐fat diet (HFD), rats exhibit varying degrees of body weight phenotypes [2]. Some rats are more prone to significant weight gain (obesity‐prone [OP]), whereas others are resistant to obesity (OR). This phenomenon was also found in mice [3]. To date, the heterogeneity between OP and OR remains largely unknown and is important in obesity research, which has been greatly advanced by the development of ‐omics approaches. In OP/OR mice, the gut microbiota profile in OP mice is distinct from that in OR mice, such as *Oscillibacter* and *Clostridium* [4], and the transfer of OP microbiota to germ‐free mice can replicate the characteristics of the OP phenotype in normal rats. This emphasizes the importance of further exploration of the microbiota genera that play key roles in the formation of OP and OR phenotypes.

Furthermore, OP‐ and OR‐related mechanisms have been explored by integrating multi‐omics data. A dysregulated gut microbiota‐bile acid axis reportedly contributes to obesity [5]. Alterations in metabolic pathways in OP rats may regulate lipid metabolism [6], and the “microbiota–gut–brain” axis may contribute to obesity resistance [4].

The microbiota may mediate the physiological adaptability of the host to influence its phenotype [7, 8, 9]. Specifically, gut microorganisms can broadly affect the body's metabolic system through metabolites they produce, including bile acids, short‐chain fatty acids, ammonia, and other bioactive compounds [10]. Notably, these metabolites can be absorbed into the enterohepatic circulation, thereby entering the circulation [11], and can induce cell signaling and proliferation, leading to alterations in physiological functions [12, 13, 14]. These processes can lead to alterations in the mucosal structure, barrier integrity, and immune activity, ultimately shaping the host phenotype.

This study aimed to investigate the relationship between microbiota–host interactions and OP and OR mice phenotypic differences to understand the dynamic changes in the gut microbiota and the related mechanisms of obesity susceptibility or resistance using an integrative approach combining microbiome, metabolomics, and transcriptomics.

## 2 RESULTS

### 2.1 Phenotypic differences in OP and OR mice

**FIGURE 1:**
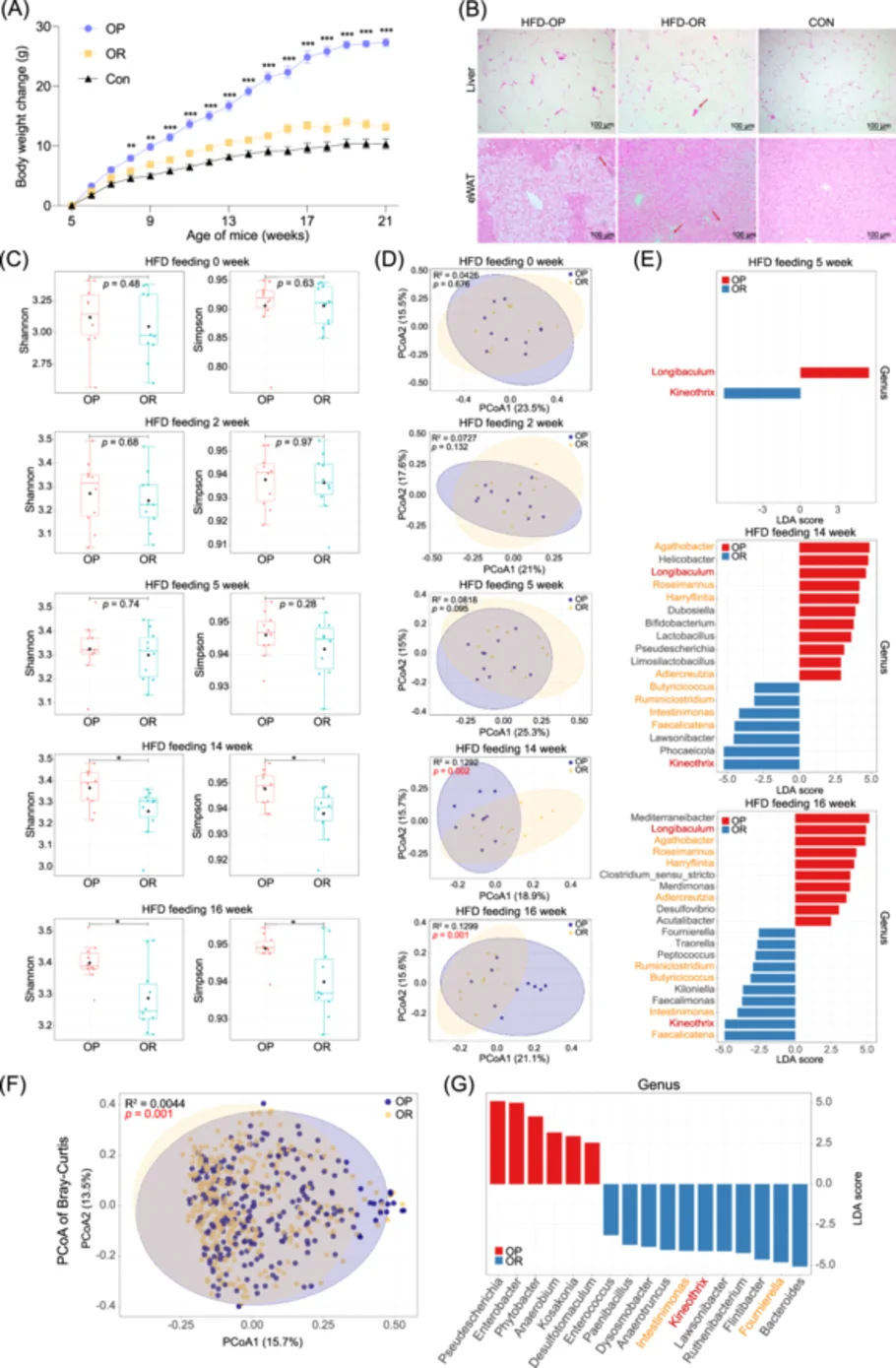
Gut microbiota changes in obesity‐prone (OP) and obesity‐resistant (OR) mice and humans. (A) Body weight change at different time points. Data are expressed as the mean ± standard error of the mean (SEM). Data were analyzed using repeated measures of a two‐way analysis of variance (ANOVA), with group and time as factors. Significant differences between groups are indicated (*p < 0.05, **p < 0.01, ***p < 0.001, OP group vs OR group, *n* = 10 in OP and OR group, *n* = 5 in Con group). (B) Representative images of hematoxylin and eosin staining of epididymal white adipose tissue (eWAT), and liver sections from the three groups. Arrows indicate the presence of adipose vesicles or inflammatory cells. Scale bars, 100 μm. eWAT: epididymal white adipose tissue. (C) The Shannon and Simpson index of fecal microbiota from different time points of the OP and OR mice (*n* = 10 samples/group), 0, 2, 5, 14, and 16 weeks of high‐fat feeding. Median (line), 1st and 3rd quartile (box margins), maximum and minimum value (whiskers). *p < 0.05 (Mann–Whitney U, *n* = 10 samples/group). (D) Principal coordinate analysis (PCoA) of fecal microbiota composition from different time points of OP and OR mice (*n* = 10 samples/group), 0, 2, 5, 14, and 16 weeks of high‐fat feeding. (E) Differences in microbial abundance between OP and OR mice fed a high‐fat diet (HFD) for 5, 14, and 16 weeks analyzed using linear discriminant analysis effect size (LEfSe). Linear discriminant analysis (LDA) effect size showing the most differentially significant abundant genus taxa enriched in microbiota from the OP and OR groups (*n* = 10 samples/group). LDA score > 2. (F) Principal coordinates analysis (PCoA) of Bray‒Curtis distances of the OP and OR humans(*p* = 0.001). Permanova analysis was used to adjust for confounders, including sex, age, smoking status, alcohol consumption, educational level, and body fat percentage. The scatter plots show principal coordinate 1 (PC1) versus PC 2, with percentages of variation explained by the components indicated. (G) LEfSe identified the microbes whose abundance was significantly different between the OP and OR humans. The genera are shown in the plot (FDR < 0.1, LDA score > 2).

Body weight changes of the mice at different stages are presented in Figure 1A. Following 3 weeks of high‐fat feeding, OP mice exhibited a significantly greater weight gain compared to OR mice (8 weeks of age; all *p* < 0.05). This difference became more pronounced as feeding time increased.

Hematoxylin and eosin (H&E)‐stained pathological sections of mouse liver and adipose tissue at 16 weeks (21 weeks of age) revealed changes in tissue structure and cell morphology (Figure 1B). Hepatocytes from OP mice showed noticeable edema, with most cells exhibiting moderate‐to‐severe extensive hepatic steatosis, mainly manifesting as macrovesicular and small vesicular steatosis. Additionally, only a small amount of lymphocyte infiltration was observed. Hepatocyte cytoplasm from OR mice exhibited a granular appearance, with small vacuoles appearing in some cells. Vacuole number was significantly less than in mice OP mice (*p* < 0.001) and more than in the normal liver (Figure S1, Table S1). Similarly, the lipid droplets in the white adipose tissue of OR mice were significantly smaller compared to those in OP mice (*p* < 0.001), yet larger than those observed in the Con group (Figure S1, Table S1).

### 2.2 Gut microbiota dynamic changes in OP and OR mice

Gut microbiota α‐ and β‐diversity of OP and OR mice was not significantly different at 0, 2, and 5 weeks of a HFD but was significantly different at 14 and 16 weeks of a HFD (*p* < 0.05) (Figure 1C,D). Key biomarkers in OP and OR mice were identified using linear discriminant analysis effect size (LEfSe) (Figure 1E). At 0 and 2 weeks of a HFD, no between‐group differences in bacterial genera were observed. After 5 weeks of a HFD, two significantly different genera, *Longibaculum* and *Kineothrix* (*p* < 0.05), appeared in OP and OR mice, respectively; we considered these as keystone bacteria. These were the first distinct genera to appear in OP and OR mice. Additionally, we validated these findings through qPCR, which confirmed the significant differences observed (Figure S2, Table S2). At 14 and 16 weeks of a HFD, *Longibaculum, Agathobacter*, *Roseimarinus*, *Harryflintia*, and *Adlercreutzia* were the dominant bacterial genera repeatedly and stably present in OP mice, whereas *Kineothrix, Butyricicoccus*, *Intestinimonas*, *Ruminiclostridium*, and *Faecalicatena* were the dominant bacterial genera repeatedly and stably present in OR mice (Figure 1E). The time points of 14 and 16 weeks of HFD feeding were chosen to represent the stage at which the phenotypes of the OP and OR groups had reached stability following long‐term dietary intervention.

### 2.3 Gut microbiota in human study

A notable variation in gut microbiota β‐diversity was identified between the OP and OR human groups (*p* < 0.05; Figure 1F). An exhaustive delineation of the baseline characteristics between the two groups is presented in Table S3. Three enriched genera, *Kineothrix*, *Intestinimonas*, and *Fournierella*, observed in OR mice, were also detected in OR humans (Figure 1G).

### 2.4 Differences in serum metabolome in OP and OR mice

**FIGURE 2:**
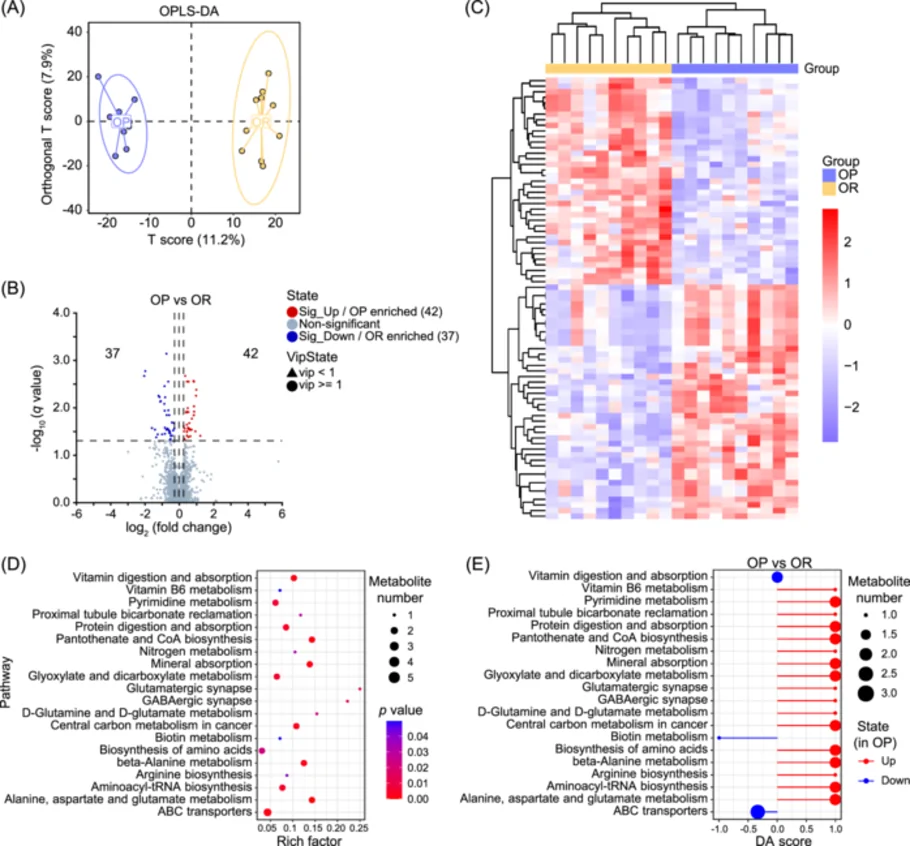
Serum metabolome changes in obesity‐prone (OP) and obesity‐resistant (OR) mice. (A) Orthogonal‐partial least square‐discriminant analysis (OPLS‐DA) of serum metabolite composition in OP and OR mice (*n* = 10 samples/group). (B) Volcano plot of serum metabolites in the two groups. Blue is down‐regulated significant differential metabolites, red is up‐regulated significant differential metabolites, and insignificant metabolites are gray (*n* = 10 samples/group). (C) Expression heatmap in the two groups. Cluster analysis of the expression levels of the differential metabolites from OP and OR mice. (D) Bubble plot of metabolic pathway Kyoto Encyclopedia of Genes and Genomes enrichment analysis of changed blood metabolites in OP and OR groups. The bubble size represents the number of differential metabolites annotated to the Pathway. (E) Differential abundance score (DA) of 20 KEGG metabolic pathways. OP versus OR. Red indicates that the expression trend of all annotated differential metabolites in this pathway is up‐regulated in OP, and blue indicates that the expression trend of all annotated differential metabolites in this pathway is down‐regulated in OP. The size of the dot at the endpoint of the line indicates the number of metabolites in the pathway, and the larger the dot, the more the number of metabolites.

In OP and OR mice, a total of 1567 serum metabolites were identified, and orthogonal partial least squares‐discriminant analysis (OPLS‐DA) modeling successfully distinguished the metabolic profiles of the two groups (Figure 2A). Integrating the results of variable projection importance (VIP), fold change, and *q*‐value in univariate analysis, 79 differential metabolites were identified between OP and OR mice, with 42 showing increased levels in OP mice and 37 exhibiting elevated levels in OR mice (Figure 2B, Table S4). Compared with OR mice, increased metabolites in OP mice included amino acids and related metabolites, organic acids, and pyridine and its derivatives; decreased metabolites included bile acids, alcohols and their derivatives, and flavonoids (Table S5). Figure 2C shows the cluster analysis results regarding the expression of differential metabolites. Significant between‐group differences in the expression of differentially expressed metabolites were visually observed using a cluster heat map.

Analysis using the Kyoto Encyclopedia of Genes and Genomes (KEGG) database revealed that the differential metabolic pathways were primarily associated with amino acid and other amino acid metabolism, cofactor and vitamin metabolism, nervous system, energy metabolism, digestive system, and carbohydrate metabolism (Figure 2D, Table S6). Compared to OR mice, the overall expression levels of 17 metabolic pathways were increased in OP mice, and overall expression levels of three metabolic pathways were decreased (Figure 2E). Amino acid metabolism had the highest proportion, including β‐alanine metabolism, d‐glutamine, and d‐glutamate metabolism, arginine biosynthesis, and alanine, aspartate, and glutamate metabolism (*p* < 0.01).

### 2.5 Differences in fecal metabolome in OP and OR mice

**FIGURE 3:**
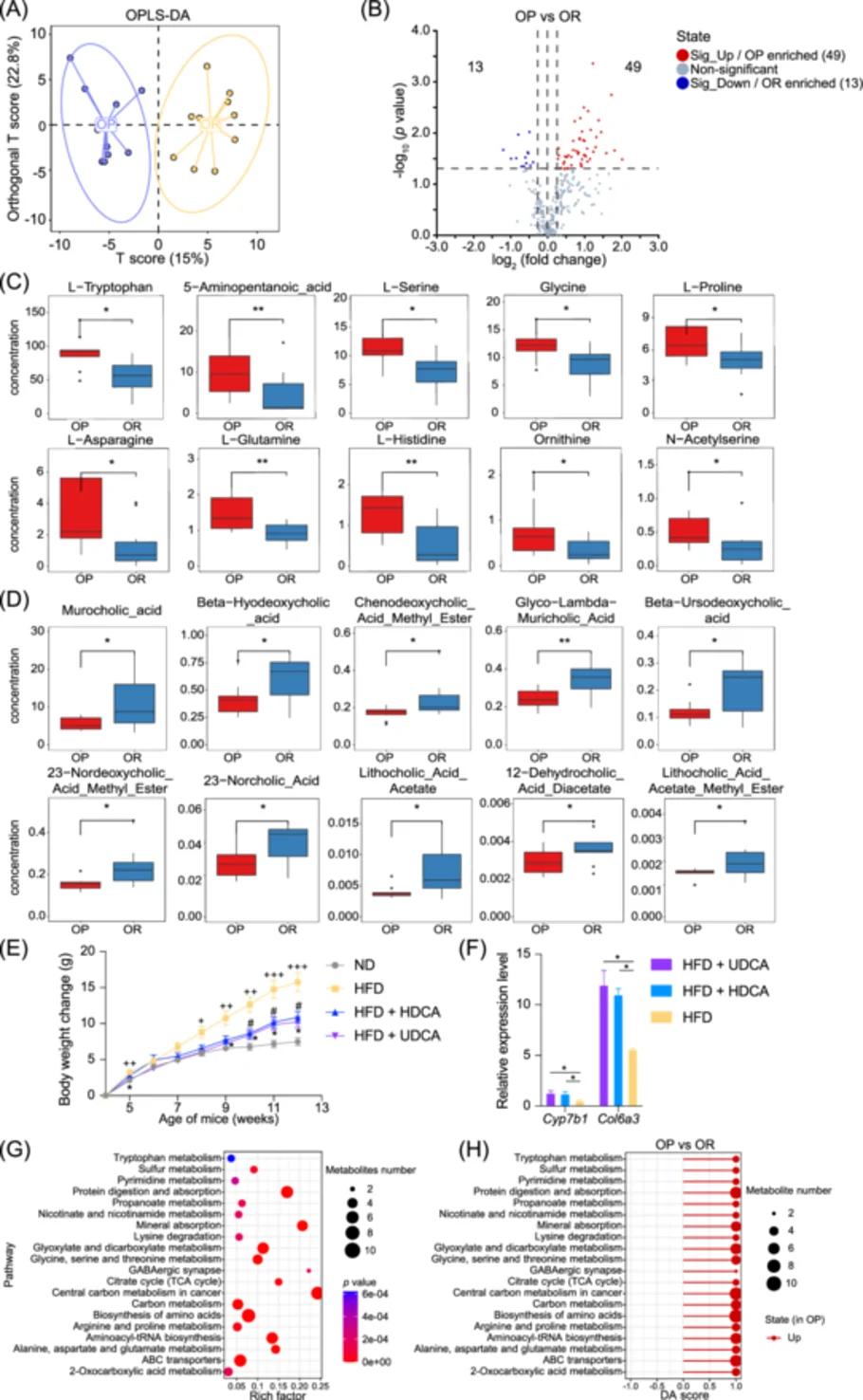
Fecal metabolome changes in obesity‐prone (OP) and obesity‐resistant (OR) mice. (A) OPLS‐DA of fecal metabolite composition in OP and OR mice (*n* = 10 samples/group). (B) Volcano plot of fecal metabolites in the two groups. Blue is down‐regulated significant differential metabolites, red is up‐regulated significant differential metabolites, and insignificant metabolites are gray (*n* = 10 samples/group). Boxplots of differential (C) amino acids and (D) bile acids in the two groups of mice. The upper whisker represents the maximum value, followed by the upper quartile (Q3), the median, the lower quartile (Q1), and the lower whisker represents the minimum value. *p < 0.05 (Welch's *t*‐test, *n* = 10 samples/group). (E) Body weight changes of mice fed a high‐fat diet supplemented with Ursodeoxycholic acid (UDCA) or Hyodeoxycholic acid (HDCA). Values represent the mean ± SEM. Data were analyzed using repeated measures of two‐way ANOVA, with group factor and time as factors. ^+^ *p* < 0.05; ^++^ *p* < 0.01; ^+++^ *p* < 0.001; ^++++^ *p* < 0.0001 (*n* = 10, HFD group vs. ND group), ^#^ *p* < 0.05 (*n* = 10, HFD + HDCA group vs HFD group), *p < 0.05; **p < 0.01 (*n* = 10, HFD + UDCA group vs. HFD group). (F) In vivo, the expression of colonic mRNAs (*Col6a3*, *Cy7b1*) was verified using quantitative real time‐polymerase chain reaction (qRT‐PCR). Values represent the mean ± SEM. *p < 0.05 (unpaired Student's *t*‐test, *n* = 3). (G) Bubble plot of metabolic pathway KEGG enrichment analysis of changed fecal metabolites in OP and OR groups. The bubble size represents the number of differential metabolites annotated to the Pathway. (H) DA of 20 KEGG metabolic pathways. OP vs. OR. Red indicates that the expression trend of all annotated differential metabolites in this pathway is up‐regulated in OP. The size of the dot at the endpoint of the line indicates the number of metabolites in the pathway, and the larger the dot, the more the number of metabolites. OPLS‐DA, orthogonal partial least squares‐discriminant analysis.

Metabolomic analysis of fecal samples was conducted, encompassing amino acids, organic acids, fatty acids, and sugars. Furthermore, over 400 small molecule metabolites, including bile acids, carnitine, phenyl and benzyl derivatives, as well as indole, were quantitatively analyzed. The OPLS‐DA model distinguished OP and OR mice based on fecal samples (Figure 3A). Overall, 62 significantly distinct metabolites were identified between OP and OR mice (Figure 3B). Of these, 22 were amino acids, including l‐tryptophan and N‐acetylserine (Figure 3C, Figure S3). Notably, levels of all these amino acids were significantly elevated in OP mice. These metabolites demonstrated robust performance (area under the curve [AUC] > 0.75) in predicting the OP metabolic phenotype in mice (Figure S3). Conversely, of the 62 metabolites, 10 were bile acids, such as β‐ursodeoxycholic acid (β‐UDCA) and β‐hyodeoxycholic acid (β‐HDCA) (Figure 3D). All bile acids were significantly elevated in OR mice. Moreover, compared to the weight changes of mice fed a HFD only, those with HDCA or UDCA for 8 weeks demonstrated a significant reduction in weight gain, accompanied by a notable upregulation in the expression of colonic mRNAs, specifically *Col6a3* and *Cyp7b1* (Figure 3E,F). Additionally, *Col6a3* and *Cyp7b1* were highly expressed in the colon transcriptomes of OR mice (Table S7, Figure S4).

Figure S5 shows the corrections for significantly different metabolites between OP and OR mice. All amino acids (marked in red) and bile acids (marked in blue) were negatively correlated. KEGG metabolic pathway enrichment analysis revealed that the differential metabolic pathways were mainly associated with amino acid metabolism, carbohydrate metabolism, digestive system, energy metabolism, and nervous system (Figure 3G, Table S8). In the overall change analysis of KEGG metabolic pathways, 20 metabolic pathways significantly increased in OP mice compared to OR mice (Figure 3H).

### 2.6 Differences in colon transcriptome in OP and OR mice

**FIGURE 4:**
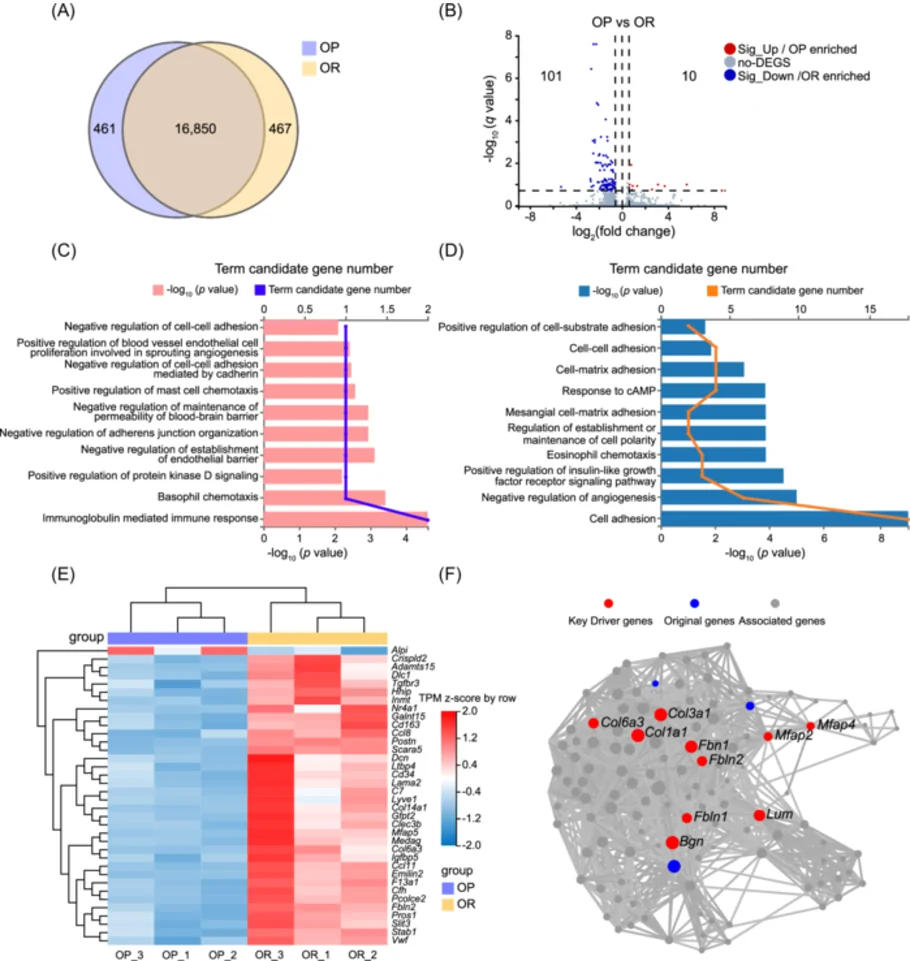
RNA‐seq analysis of the colon in obesity‐prone (OP) and obesity‐resistant (OR) mice. (A) Venn diagram of the numbers of genes in the OP and OR groups. The two groups have 16,850 shared genes, with 461 and 467 unique genes in the OP and OR groups, respectively. (B) Volcano plot of differentially expressed genes (DEGs) between OP and OR mice. Fold change ≥ 1.2 or fold change ≤ 0.83 & *q*‐value < 0.2. (C, D) Gene Ontology (GO) functional enrichment analysis of differentially expressed genes. (E) Hierarchical clustering shows 36 differentially expressed genes between OP and OR mice. Fold change ≥ 1.2 or fold change ≤ 0.83 & *q*‐value < 0.05. (F) Graphical view of 10 key driver genes. The size of the nodes represents the number of connections a gene has with other genes in the network.

To investigate the potential mechanisms underlying colonic core bacterial interference or protection in the host, RNA sequencing (RNA‐seq) analysis was conducted to quantify colonic gene expression profiles in OP and OR mice. A total of 17,778 genes were detected (Figure 4A), and Gene Ontology (GO) enrichment analysis was conducted on the differentially expressed genes, defined by a fold change ≥1.2 or ≤0.83 and a *q*‐value < 0.2. Among the total differentially expressed genes, 10 and 101 differentially expressed mRNAs (DEmRNAs) were upregulated and downregulated in OP mice, respectively, compared with OR mice (Figure 4B). GO analysis revealed enrichment of terms related to biological processes associated with immunity and intestinal structure in OP mice: negative regulation of the establishment of the endothelial barrier, negative regulation of the adherens junction organization, negative regulation of cell–cell adhesion mediated by cadherin, and negative regulation of cell–cell adhesion (Figure 4C). In contrast, cell adhesion, regulation of establishment or maintenance of cell polarity, mesangial cell–matrix adhesion, cell–matrix adhesion, cell–cell adhesion, and positive regulation of cell–substrate adhesion were enriched in OR mice (Figure 4D).

Through further differential gene screening, based on criteria of fold change ≥1.2 or ≤0.83 and *q*‐value < 0.05), 36 differentially expressed mRNAs were screened in OP and OR mice (Figure 4E). Ten key driver genes were identified based on 36 DEmRNAs genes using key driver analysis (KDA), which are crucial regulators within the gene networks of OP and OR mice (Figure 4F, Table S7). The most significant key driver genes, including *Fbln2*, *Col6a3*, *Col3a1*, *Col1a1*, and *Bgn*, exhibited significantly higher expression levels in OR mice compared to OP mice, with *p* values of 8e‐8, 4.17e‐5, 1.50e‐4, 2.18e‐4, and 9.20e‐4, respectively.

**FIGURE 5:**
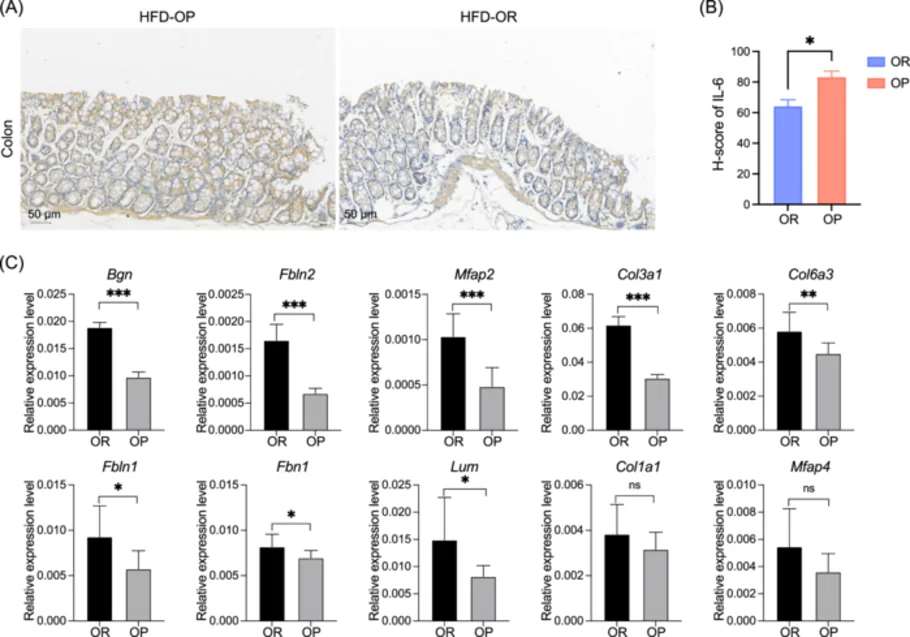
Validation of intestinal barrier responses based on transcriptomic data. (A) The expression of Interleukin‐6 (IL‐6) in colon tissues was detected by immunohistochemical analysis Scale bars, 50 μm. (B) H‐score of IL‐6 immunoreactivity in obesity‐prone (OP) and obesity‐resistant (OR) groups. *OP versus OR group; Values represent the mean ± SEM. *p < 0.05 (unpaired, two‐tailed *t*‐test, *n* = 3. (C) Quantitative real time‐polymerase chain reaction validates 10 key colon barrier driver genes in OP and OR mice. Values represent the mean ± SEM. *p < 0.05; **p < 0.01; ***p < 0.001 (unpaired, two‐tailed *t* test, *n* = 3).

Inflammatory changes in colon tissue were evaluated by tissue expression of Interleukin‐6 (IL‐6) identified by immunohistochemical (Figure 5A). The immunoreactivity of IL‐6 in OP and OR mice examined was quantified by the H‐score system, with the findings presented in Figure 5B. The IL‐6 immunoreactivity H‐score was significantly elevated in the OP group compared to the OR group (*p* < 0.05). The data indicated that the OP mice exhibited a more pronounced inflammatory response than the OR mice. Quantitative real time‐polymerase chain reaction (qRT‐PCR) was employed to validate the expression of 10 key driver genes, revealing that 8 of these genes were significantly upregulated in OR mice compared to OP mice (Figure 5C).

### 2.7 Correlations among microbiome, metabolome, and transcriptome

**FIGURE 6:**
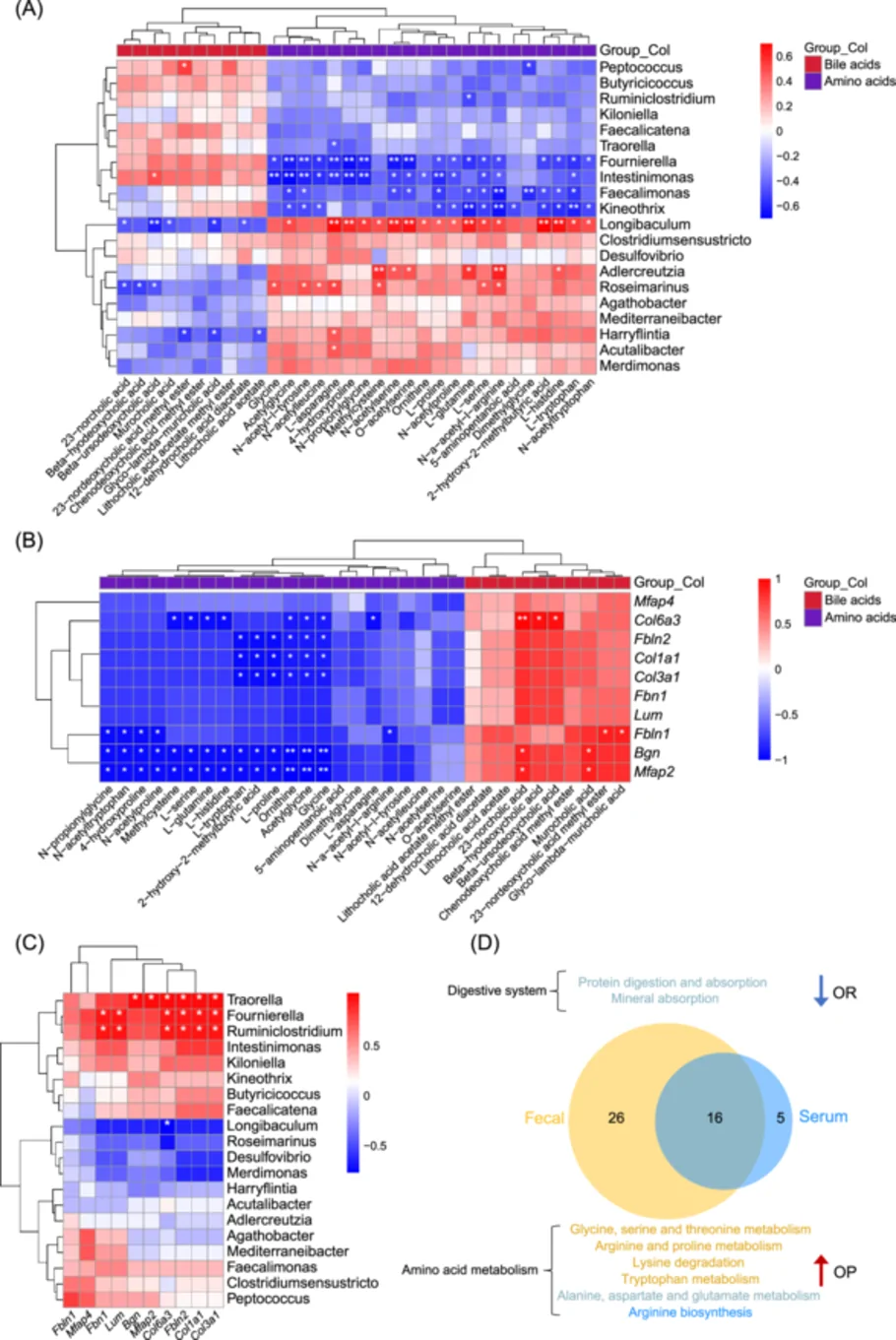
Correlation analysis amongst microbiome, metabolome, and transcriptome. (A) Spearman's correlation between gut microbiota and differential metabolites in fecal at the genus level. *X* and *Y* axes are differential metabolites and genera, respectively. The value of *p* < 0.05 is marked with “*,” and *p* < 0.01 is marked with “**.” (B) Combined metabolome and transcriptome analysis. *X* and *Y* axes are differential metabolites in fecal and driver genes, respectively. The value of *p* < 0.05 is marked with “*,” and *p* < 0.01 is marked with “**.” (C) Spearman's correlation of genus‐level gut microbiota with 10 key driver genes. X and Y axes are driver genes and differential genera, respectively. The value of *p* < 0.05 is marked with “*,” and *p* < 0.01 is marked with “**.” (D) Venn diagram of the numbers of Go terms in the OP and OR groups. The color of the pathway name is consistent with the group represented by the color of the group in the Venn diagram, that is, the yellow pathway represents the fecal group, the blue pathway represents the serum group, and the green pathway represents the overlap of the two groups. OP, obesity‐prone; OR, obesity‐resistan.

Correlation analysis between potential metabolites and dominant gut microbiota at the genus level identified associations between 20 gut bacterial genera and 32 metabolites. *Kineothrix*, *Butyricicoccus*, *Intestinimonas*, *Rumiclostridium*, and *Faecalicatena* exhibited negatively correlated with amino acid metabolites while showing positive correlations with bile acid metabolites. In contrast, *Longibaculum, Butyricicoccus, Intestinimonas, Rumiclostridium*, and *Faecalicatena* demonstrated positive correlations with amino acid metabolites and negative correlations with bile acid metabolites (Figure 6A).

Correlation coefficients between fecal metabolites and driver genes involved in cell adhesion, migration, and extracellular matrix structure are shown in Figure 6B. Bile acid metabolites were positively correlated with intestinal driver genes (*Col6a3*, *Fbln1*, *Bgn*, and *Mfap2*), whereas amino acid metabolites were negatively correlated with intestinal mucosal barrier driver genes (*Col6a3*, *Fbln2*, *Col1a1*, *Col3a1*, *Fbln1*, *Bgn*, and *Mfap2*).

Figure 6C shows correlation coefficients between genus‐level gut microbiota and driver genes involved in cell adhesion, migration, and the structure of the extracellular matrix. *Longibaculum* was significantly negatively correlated with *Col6a3*, whereas *Traorella, Fournierella*, and *Rumiclostridium* were significantly positively correlated with *Fbn1*, *Lum*, *Bgn*, *Mfap2*, *Col6a3*, *Fbln2*, *Col1a1*, and *Col3a1*.

An integrated analysis of the serum and fecal metabolomes was performed, and 42 and 21 KEGG pathways were altered in fecal and serum metabolomes, respectively (Figure 6D). Among these, six pathways related to amino acid metabolism exhibited greater enrichment in OP mice compard to OR mice. Moreover, the two pathways associated with the digestive system were less enriched in OR mice compared to OP mice. Notably, 16 KEGG pathways overlapped between the fecal and serum metabolomes (Table S9).

## 3 DISCUSSION

We investigated the relationships between OP and OR phenotypic differences and host–microbiota interactions using microbiota sequencing, metabolomics, and transcriptomic analyses, as summarized in the graphical abstract. Our study demonstrated that *Longibaculum* and *Kineotrix* might served as keystone bacteria for dynamic changes in gut microbiota in OP and OR mice, respectively, potentially instigating community effects in gut microbiota of the two groups. Augmentation of beneficial microbiota, an increase in non‐12‐OH bile acid metabolites, and fortification of the intestinal barrier were robustly correlated with an anti‐obesity phenotype. The *Longibaculum*‐UDCA‐*Col6a3* pathway has been implicated as a potential underlying mechanism. Impairment of the intestinal mucosal barrier and bacterial translocation could intensify weight gain, whereas disparities or elevations in the amino acid profile could act as predictive indicators of metabolic phenotypes that are predisposed to obesity.

We first analyzed dynamic changes in gut microbiota during the development of OP and OR mice and obtained the core dominant bacterial genera. Notably, phenotypic differences in obesity are a manifestation of polymicrobial involvement, where various gut microbial factors and metabolic disorders coalesce to cause abnormal host–microbe interactions [15, 16]. However, no reports exist on the changes in the microbiome during the formation of OP and OR in mice. Our study revealed that *Longibaculum* and *Kineothrix* were keystone bacterial genera in OP and OR mice, respectively. Notably, the *Kineothrix* genus was significantly elevated not only in OR mice but also in OR humans. *Kineothrix alysoides*, a species belonging to the genus *Kineothrix* [17], significantly increased by 57‐fold and improved intestinal barrier function following treatment in an animal model of fatty liver [18]. Our transcriptome results revealed that, compared to OP mice, OR mice showed significant enrichment of intestinal cell adhesion functional pathways, including positive regulation of cell adhesion and cell–matrix adhesion.

Moreover, after 14 and 16 weeks of high‐fat feeding, other bacterial genera that stably exist in OR mice, with *Kineothrix* as the primary driver of OR phenotype formation, included *Butyricicoccus* [19, 20], *Intestinimonas* [21], *Rumiclostridium* [22], and *Faecalicatena* [23], aligning with prior studies on microbial alterations associated with weight loss in mice or humans. The functions of these four beneficial bacteria (*Butyricicoccus* [24], *Intestinimonas* [25], *Rumiclostridium* [26, 27], and *Faecalicatena* [28]) are similar to those of *Kineothrix*, contributing significantly to the production of short‐chain fatty acids (SCFAs). The butyrate‐producing bacterium *Butyricicoccus* interacts synergistically with other beneficial bacteria to decrease the *Firmicutes* to *Bacteroidetes* (F/B) ratio. Notably, our study revealed that the F/B ratio in OR mice was significantly reduced compared to OP mice, indicating superior intestinal homeostasis in OR mice (Figure S6). A substantial increase in the F/B ratio in OP mice is considered dysbiosis [29], which promotes lipid production, abnormal weight gain, and the development of chronic metabolic diseases [30, 31]. *Butyricicoccus*, a bacterium that protects the intestinal barrier, plays a crucial role in intestinal homeostasis [32] by ensuring mucus production [33] and maintaining tight junction integrity [34]. Oral administration of *Butyricicoccus* can reduce intestinal inflammation in rats with colitis [35]. A high abundance of *Butyricicoccus* in patients with obesity contributes to body mass index (BMI) reduction during weight loss treatments [35], suggesting its potential role in promoting an anti‐obesity metabolic phenotype. *Intestinimonas*, which was also highly expressed in our OR metabolic phenotype, converts amino acids into butyrate in the intestine [21]. We found that 22 amino acids were significantly reduced in OR mice compared to OP mice. Notably, several amino acids exhibited a significant negative correlation with *Intestinimonas*, indicating its critical role in regulating protein balance [36] and maintaining intestinal homeostasis in OR mice. Intestinal microbiota disorder and decrease in *Intestinimonas* abundance in OP mice were alleviated by supplements, thereby mitigating the metabolic state of OP mice [37]. *Ruminiclostridium* showed a strong negative correlation with most metabolic parameters [38], such as intestinal permeability, blood lipids [39], glucose, and insulin. The key driver genes analyzed to maintain intestinal immunity and mechanical barriers were significantly positively correlated with *Ruminiclostridium* upregulation in OR mice. Additionally, *Ruminiclostridium* is involved in regulating lipid metabolism [40] and the promotion of beige adipocyte development within white adipose tissue [41], implying that *Ruminiclostridium* may reduce body weight by affecting energy metabolism metabolites. All of these stable existing communities associated with the OR phenotype essentially produce SCFAs, which are beneficial for maintaining the function and structure of intestinal epithelial cells while regulating the balance of the intestinal microbiota. These results suggested that the shaping of the obesity‐resistant phenotype of OR mice by the intestinal microbiome may be a result of community effects. Therefore, the OR phenotype of mice was closely related to beneficial intestinal commensals, i.e., *Butyricicoccus*, *Intestinimonas, Rumiclostridium*, and *Faecalicatena*, headed by *Kineothrix*. In contrast, the stable keystone bacterium *Longibaculum* in OP mice showed a significant negative correlation with *Col6a3*, a driver gene that maintains the intestinal mechanical barrier, and with the intestinal mucosal integrity index [42]. *Longibaculum muris*, a species of this genus, showed an increased abundance in mice consuming a high‐protein diet [43], and its fecal levels were positively associated with oral glucose intolerance in rats [44]. However, the potential pathways by which *Longibaculum* affects its functions remain unclear. We observed that other bacterial genera that stably coexisted with *Longibaculum* in OP mice after 14 and 16 weeks of high‐fat feeding contributed to the formation of the OP phenotype, including *Harryflintia* [45], *Adlercreutzia* [46], *Agathobacter* [47, 48], and *Roseimarinus*. These bacterial changes were consistent with previous reports on microbial alterations in overweight mice and humans. These detrimental bacteria (*Harryflintia* [49, 50], *Adlercreutzia* [46, 51], and *Agathobacter* [47]) were positively correlated with metabolic markers, such as body weight, inflammation, fat mass, and glucose levels, which are implicated in obesity and related metabolic disorders. Moreover, *Harryflintia* demonstrated a negative association with certain markers related to intestinal barrier integrity and mucus function [50]. The positive correlation between *Adlercreutzia* and body weight was prospective [52], indicating a bi‐directional causal relationship between *Adlercreutzia* and obesity. The proportion of *Agathobacter* was positively correlated with the rapid development of overweight and obesity in children aged 1‒2.5 years [53]. Although the relationship between *Roseimarinus* and obesity has rarely been studied, it may serve as a novel marker of obesity. The elevated abundance of these bacterial genera is a common feature of obesity‐related dysbiosis, suggesting that they act as potentially harmful microbiota and contribute to the development of the OP phenotype. We inferred that the OP phenotype was closely related to harmful intestinal symbionts, i.e., *Agathobacter*, *Roseimarinus*, *Harryflintia*, and *Adlercreutzia*) led by *Longibaculum*. Therefore, we hypothesized that an increase in beneficial bacteria in the intestine could counteract obesity after an HFD, whereas the disturbance of the intestinal microbiota, metabolic abnormalities, and weakened intestinal mucosal barrier exacerbate weight gain.

Gut microbiota produces various metabolites through the anaerobic fermentation of undigested dietary components that reach the colon and endogenous substances derived from microorganisms and the host [54]. These microbial metabolites can penetrate host cells, interacting directly to impact epithelial cells and mucosal barriers, or enter the portal blood and influence systemic metabolic health [55]. Our study revealed strong correlations between colonic microbiota and metabolites by integrating metabolomics with microbiota‐sequencing analysis, demonstrating that changes in levels of bile acids, amino acids, SCFA, indole derivatives, and other metabolites may underlie the phenotypic distinctions between OP and OR.

Our study found a significant elevation of non‐12‐OH bile acids (muricholic acid [MCA], UDCA, HDCA, lithocholic acid [LCA], chenodeoxycholic Acid [CDCA], and derivatives of other alternative pathways, in OR mice compared to OP mice. Furthermore, 16S rDNA gene sequencing analysis using PICRUSt revealed a significant enrichment of bile acid metabolism pathways in OR mice (Figure S7). CYP7B1, a critical enzyme in the bile acid alternative pathway [56], was significantly upregulated in OR mice, indicating activation of the bile acid alternative pathway, which was also related to bacterial suppression with a high abundance of bile salt hydrolase (BSH) genes. In the present study, the BSH‐rich bacteria [57] (*Bacteroides*, *Lactobacillus*, *Bifidobacterium*, and *Clostridium*) in OR mice were inhibited, leading to conjugated bile acid accumulation in the distal colon [58], comprising non‐12‐OH bile acids. *Longibaculum* was also a genus with the BSH gene (https://www.ncbi.nlm.nih.gov/nuccore/2428088975). These findings indicate that gut microbiota dysbiosis contributes significantly to the weight‐gain‐resistant metabolic phenotype by regulating the alternative bile acid pathway. Moreover, our intervention study showed that HFD with UDCA or HDCA (non‐12‐OH) supplementation inhibited weight gain in mice, further confirming the role of alternative bile acid synthesis pathways. It has been reported UDCA or HDCA supplementation can reduce metabolic dysfunction by restoring intestinal barrier integrity [59]. In addition, our analysis revealed that UDCA and *Col6a3* (a key driver gene of intestinal barrier integrity) were significantly positively correlated, whereas UDCA and *Longibaculum* were significantly negatively correlated, suggesting that the inhibition of bacteria with BSH genes (*Longibaculum*) activates secondary bile acids (such as UDCA) to maintain intestinal barrier stability by upregulating genes involved in cell adhesion and migration (such as *Col6a3*) and helps ameliorate weight gain.

Amino acid profiles are potential biomarkers of obesity susceptibility and have been suggested as useful markers for early intervention in childhood obesity [60]. However, further animal studies are unavailable. The current study identified 22 gut amino acid metabolites and six amino acid metabolism pathways that were significantly overexpressed in OP mice; elevated amino acid levels are associated with intestinal microbiota dysbiosis [61]. *Bacteroides vulgaris* was identified as the primary species mediating the link between specific amino acid biosynthesis and insulin resistance in HFD‐fed mice [62, 63, 64, 65, 66]. The serum metabolome of individuals with insulin resistance exhibited increased concentrations of specific amino acids [66]. Improvements in insulin resistance and glucose metabolism following a high‐fiber diet have also been associated with an increased *Prevotella‐*to*‐Bacteroides* ratio [67, 68]. Gut microbiota rich in *Prevotella* aids weight loss [62, 63, 64, 65] and cholesterol reduction [69]. Our study corroborates this hypothesis. The overexpression of *Prevotellaceae*, *Prevotellamassilia*, *Prevotella/Bacteroides* ratio, intestinal amino acid profile, and suppression of the expression of six amino acid pathways reflect the metabolic phenotype of OR mice (Figure S6). Excess colonic amino acids can also influence intestinal microbiota [70], promoting the growth of harmful bacteria such as *Bacteroides*, *Clostridium*, and *Helicobacter pylori*. This indicates that the interaction between amino acid imbalance and intestinal bacteria significantly contributes to obesity‐prone metabolic states. Additionally, in a 12‐year follow‐up study, Wang et al. [71] underscored the potential importance of amino acid metabolism in early diabetes onset and suggested that amino acid profiles contribute to diabetes risk assessment and may serve as predictors of future diabetes in individuals without diabetes. Other studies have demonstrated significant positive correlations between plasma levels of specific amino acids and both BMI [72] and glucose resistance [73]. Our findings highlight the potential utility of amino acid profiles as markers of obesity susceptibility, evidenced by the overexpression of amino acid metabolic pathways in both the blood and feces of OP mice [74]. Therefore, our study indicates that an imbalance or increase in obesity‐related amino acids may exacerbate the OP phenotype, whereas the maintenance of obesity‐related bile acid metabolism supports OR, with the intestinal microbiota serving as a critical regulatory factor.

The gut barrier function encompasses three primary barriers: mechanical, ecological, and immunological [75]. Disorganization of the ecological barrier, characterized by microbial dysbiosis, frequently results in dysfunction of the mechanical and immunological barrier [76] and causes inflammation [77]. To investigate the potential association between gut microbiota and host immune and intestinal barrier responses in OP and OR mice, RNA‐seq analysis was conducted on colonic samples. The colon harbors the most densely populated and metabolically active microbial community in the gut, consisting of over 10^13^ individual microbial cells [78]. GO analysis in our study suggested that intestinal cell adhesion was disrupted in OP mice. The compromised integrity of the intestinal barrier allows commensal microbiota to access intestinal epithelial cells [79]. In the presence of intestinal barrier damage, the OP phenotype may be associated with an increase in potentially pathogenic microorganisms and/or bacterial community components, such as *Bacteroidetes*, *Clostridium*, and *Helicobacter pylori*, triggering a chronic inflammatory response [80]. This was confirmed by the immunohistochemical, which showed increased inflammatory cells in OP mice compared to OR mice. Based on GO enrichment results, key driver genes (*Col6a3*, *Bgn*, *Fbln2*, *Mfap2*, *Col3a1*, *Fbln1*, *Fbn1*, and *Lum*) involved in cell adhesion, migration, and extracellular matrix structure were significantly upregulated in OR mice, which was validated using real‐time PCR. Most previous studies have reported reduced expression of these genes in adipose tissue of obese mice or humans [81], and our study confirms reduced colonic expression of these genes in OP mice. Summarily, impaired intestinal mucosal barrier and bacterial translocation may aggravate weight gain, whereas intestinal homeostasis and barrier integrity are maintained in OR mice.

Our study focuses on the regulatory role of host gene expression in the colon, which hosts the most diverse and abundant microbiota. The colon is central to complex fermentation processes, producing metabolites such as bile acid derivatives, amino acid metabolites, and SCFAs. These aspects are central to our metabolomic and microbiome analyses and provide valuable resources for further investigations, such as fecal microbiota transplantation experiments and cell‐based studies. However, significant differences in microbial composition and metabolite profiles across different gut regions highlight the need for future exploration of metabolomics and microbiomes throughout the gastrointestinal tract.

## 4 CONCLUSION

Our results showed that keystone bacteria can potentially drive community effects on microbiota dynamics. Impairment of the intestinal mucosal barrier, bacterial translocation, and elevated levels of obesity‐associated amino acids could potentially exacerbate weight gain. Conversely, gut microbiota of OR mice preserved homeostasis, elevated non‐12‐OH bile acids, and maintained intestinal barrier integrity. The importance of the *Longibaculum*‐UDCA‐*Col6a3* pathway was also emphasized. These findings could aid in identifying crucial obesity‐associated intestinal microbial markers and potential anti‐obesity targets, providing a valuable resource for advancing the understanding of obesity‐prone and obesity‐resistant phenotypes.

## 5 METHODS

### 5.1 Animal study design

A total of 87 male C57BL/6J mice (4 weeks old, 17‒19 g, specific pathogen‐free grade) were purchased from Shanghai Slack Laboratory Animal Co., Ltd. and acclimated for 1 week at the Zhejiang University Animal Experimental Center. Of these, 47 mice (*n* = 47) were randomly assigned to two dietary groups: the (1) normal diet (13% fat, *n* = 5, Jiangsu Xietong Pharmaceutical Bio‐engineering Co., Ltd) and (2) HFD (60% fat, D12492, *n* = 42, Trophic Animal Feed High‐Tech Co., Ltd) groups [82], and fed for 16 weeks. Fresh fecal samples were obtained from mice at 0, 2, 5, 14, and 16 weeks of feeding (corresponding to 5, 7, 10, 19, and 21 weeks of age, respectively) for subsequent intestinal microbiome genomic analysis. Metabolomics analysis was based on fresh fecal samples collected from mice at 16 weeks of feeding. Mice were anesthetized (intraperitoneally injected with 1% *pentobarbital sodium*) at 16 weeks of feeding or 21 weeks of age, and approximately 0.5–1 mL of blood was obtained. The serum was separated by centrifuging the blood at 3000 rpm for 10 min. Sections of the liver, colon, and white adipose tissues were harvested after euthanasia. Tissue samples were stored at −80°C until analysis, and another portion of tissue samples was fixed in tissue fixative and embedded in paraffin blocks for preservation.

To examine the effect of HDCA and UDCA supplementation on weight, another 40 mice were randomly allocated to four dietary groups: the normal diet (*n* = 10) (13% fat), HFD (*n* = 10) (60% fat), HFD + 0.5% UDCA (*n* = 10) (60% fat), and HFD + HDCA (*n* = 10) (60% fat) groups. Body weight was monitored on a weekly basis throughout the duration of the experiment. The mice were anesthetized at 8 weeks of feeding or 12 weeks of age, and colonic sections were collected.

All mice were maintained under specific pathogen‐free conditions with ad libitum access to food and water in a controlled environment (temperature 20°C‒22°C, humidity 45 ± 5%, 12 h light/dark cycle). This study was approved by the Zhejiang University Ethics Committee and followed the guidelines of the Zhejiang University Experimental Animal Center (approval number: ZJU20220424).

### 5.2 Definition of OP and OR mice

At 16 weeks of feeding or 21 weeks of age, 42 mice in the HFD feeding group were classified into two subgroups based on their weight gain during HFD feeding: the top 10 obese mice were defined as OP (the upper quartile), and the bottom 10 mice were defined as OR (the lower quartile) [82]. The remaining 22 mice were excluded from the analysis.

### 5.3 Human study design

Study participants were recruited from the Lanxi Cohort, a community‐based cohort study conducted between June and August 2019 in urban areas of Lanxi City, Zhejiang Province, China [83]. Detailed information regarding the participants' inclusion and exclusion criteria is provided in Supplementary Information, Method S1 [84]. Overall, 586 participants with high energy intake were included. Participants with BMI ≥ 24 kg/m^2^ were defined as the OP group (*n* = 248), whereas those with BMI < 24 kg/m^2^ were defined as the OR group (*n* = 338). Baseline information, including sex, age, smoking and drinking status, educational level, body fat percentage, physical activity level, and biochemical data, was obtained through a face‐to‐face questionnaire survey and physical examinations. Fecal samples were also collected on the cohort survey day for each participant and stored at −80°C until analysis. Continuous variables are expressed as the mean and standard deviation (SD) or standard error of the mean. Categorical variables are expressed as percentages (%). This study was approved by the Ethics Committee of the School of Public Health at Zhejiang University (ZGL201905‐1).

### 5.4 Microbiome sequencing and data analysis in fecal samples

#### 5.4.1 DNA extraction

Total genomic DNA was extracted from fecal samples using the DNeasy® PowerSoil® Kit (Qiagen), following the manufacturer's instructions. Subsequently, the DNA concentration and integrity of the extracted genomic DNA were determined using Qubit and 1% agarose gel electrophoresis, respectively.

#### 5.4.2 PCR amplification and microbiota sequencing

The V3‒V4 region of the bacterial 16S rDNA gene was amplified using the primers 341F (5ʹ‐CCTACGGGNGGCWGCAG‐3ʹ) and 805R (5ʹ‐GACTACHVGGGTATCTAATCC‐3ʹ) combined with barcode sequences. Amplicon pools were prepared for sequencing, and the size and quantity of the amplicon library were assessed using an Agilent 2100 Bioanalyzer (Agilent) and Library Quantification Kit for Illumina (Kapa Biosciences), respectively. Libraries were sequenced on a NovaSeq PE250 platform.

#### 5.4.3 Data analysis

Liu et al. proposed a reproducible “analysis pipeline” using the R language, which refers to a particular script that combines dozens of software programs organically for complex analysis tasks [85]. We used the analysis pipeline to convert raw reads (fastq format) into amplicon sequence variants (ASVs) table. ASVs were sequences with 100% similarity.

ASV abundance matrix and annotation results served as the foundation for subsequent biological information analysis, which included diversity analysis (*α*, *β*), species composition, and LEfSe, conducted on OP and OR mice and OP and OR humans. The Mann–Whitney *U* test, a rank‐sum test, was used to examine disparities in the alpha diversity index (Shannon and Simpson indices) between groups. To analyze beta diversity across distinct groups, the Bray‒Curtis distance was employed as a metric to encapsulate the variance between communities, where principal coordinate analysis was performed. To analyze differences between the two groups, LEfSe analysis was performed using the website (http://huttenhower.sph.harvard.edu/galaxy/), with *p* < 0.05 and a linear discriminant analysis (LDA) score > 2.0. Correlations between bacteria, metabolites, and genes were calculated using Spearman's rank correlation coefficients. Furthermore, additional analyses using multivariable linear regression and Permanova were conducted for the OP and OR human groups to adjust for factors such as sex, age, smoking status, alcohol consumption, education level, and body fat percentage.

### 5.5 Metabolome measurements and data analysis in serum and fecal samples

#### 5.5.1 Serum sample preparation

Serum samples were thawed on ice, and serum metabolites were extracted with an extraction agent containing internal standard 1 (methanol:acetonitrile:water = 4:2:1, v/v) and 50% methanol buffer. All samples were analyzed via liquid chromatography‐mass spectrometry (LC‐MS) using a Waters 2777C Ultra Performance Liquid Chromatography (UPLC) system (Waters) coupled with a Q Exactive HF high‐resolution mass spectrometer (Thermo Fisher Scientific), according to the manufacturer's instructions. Metabolites were separated and detected using UPLC‐MS.

#### 5.5.2 Fecal sample preparation

Gut metabolites were extracted from fecal samples thawed on ice in a 50% methanol buffer. The samples, quality control, and standard music were derivatized and analyzed using LC‐MS and a sample diluent according to the manufacturer's instructions. A Waters ACQUITY UPLC I‐Class Plus (Waters) coupled with a QTRAP6500 Plus high‐sensitivity mass spectrometer (SCIEX) was used for metabolite separation and quantification. Each multiple reaction monitoring transition (ion transition) was identified and integrated using HMQuant software. Metabolite peaks were extracted and identified from the original mass spectrometry data, and information, including molecular weight, retention time, peak area, and identification results of the metabolites, was obtained.

#### 5.5.3 Data analysis

Data acquired by the instrument were preprocessed using MetaX software and then analyzed, identified, and annotated using the Human Metabolome Database and KEGG to explore the classification characteristics and functional attributes of the KEGG metabolic pathways of different metabolites. To screen for group differences, data were first log2 transformed, and PLS‐DA models were established between the experimental groups. The Pareto method was used for scaling. An orthogonal signal correction was applied to perform OPLS‐DA between the experimental groups. This reduced the model's complexity and enhanced the model's explanatory ability while ensuring its prediction ability. Additionally, the VIP was calculated to evaluate the influence and explanatory ability of each metabolite expression pattern on sample classification discrimination. Metabolites with VIP > 1 were considered helpful in distinguishing sample categories. The difference analysis also included conventional univariate analysis methods, such as fold‐change analysis and t‐tests. Using the Welch's *t*‐test, the significance of the expression level of each metabolite in each comparison group was tested to obtain a *p*‐value. The *p*‐value was corrected using the Benjamini‒Hochberg algorithm to obtain the *q*‐value. The *q*‐value was used to assess the significance of the differences between the two groups of samples. The fold change reflected whether the mean value of a metabolite in the two groups of samples changed, and the *q*‐value reflected whether this change was statistically significant. Metabolites with a fold change of ≥1.2 or ≤0.83 and *p*‐value < 0.05 were considered significantly different. Receiver operating characteristic (ROC) curve analysis was performed on the differential metabolites. In ROC analysis, an AUC > 0.75 indicated the metabolite's ability to serve as a biomarker.

### 5.6 Transcriptome RNA sequencing and data analysis in colon samples

Total RNA was extracted and purified using TRIzol reagent (Invitrogen) according to the manufacturer's instructions. mRNA was enriched from the total RNA, and a strand‐specific transcriptome library was constructed and sequenced using the DNBSEQ high‐throughput platform. Raw sequencing data were filtered using SOAPnuke (v1.5.6) [86] to obtain clean data. Dr Tom's multi‐omics data‐mining system (https://biosys.bgi.com) was used for data mining and mapping. Differential gene analysis was performed using Bowtie2 (v2.3.4.3) [87] to align the clean data with the reference gene set (GCF_000001635.27_GRCm39). RSEM [88] (v1.3.1) software was used to quantify gene expression, and pheatmap (v1.0.8) [89] was used to generate clustering heatmaps of gene expression levels in different samples. DESeq. 2 (v1.4.5) [89] was used to detect differentially expressed genes between OP and OR mice, with fold‐change criteria of ≥1.2 or ≤0.83 and a *q*‐value < 0.2. To further explore gene functions related to phenotypic changes, GO (http://www.geneontology.org) and KEGG (https://www.kegg.jp/) enrichment analyses were performed on differentially expressed genes based on hypergeometric testing using Phyper (https://en.wikipedia.org/wiki/Hypergeometric_distribution), with a *q*‐value ≤ 0.05 as the threshold for significant enrichment in candidate genes. To identify the key driver genes among the selected genes, we performed key‐driver‐gene analysis (KDA) based on the scores of the STRING11 database, with a cutoff point of >500 (BGI's Dr.Tom system).

### 5.7 H&E staining

For histological analysis, the white adipose tissue, and liver tissue were fixed in 4% paraformaldehyde, embedded in paraffin, sectioned, and stained with H&E. The size of the lipid droplets, degree and extent of inflammation, and severity and extent of mucosal and crypt damage were assessed using a microscope (Olympus) at 100× magnification. Images of each section were captured. The vacuolation area of eWAT cells and liver cells was quantified using ImageJ 1.54g software (National Institutes of Health).

### 5.8 Immunohistochemistry staining and scoring

The colon tissue sections were initially embedded in xylene‐dewaxed paraffin and subsequently rehydrated with a graded ethanol series. Following the exhaustion of endogenous peroxidase and the blocking of antigens, sections were incubated with primary antibodies against IL‐6 (1:200, Servicebio) overnight at 4°C. Subsequently, the sections were incubated with secondary antibodies (AiFang Biological) for 30 min at room temperature. Subsequently, the staining was developed using Diaminobenzidine, and the tissues were counterstained with hematoxylin.

For immunohistochemical analysis, the slides were imaged using a light microscope (Nikon E100 with DS‐U3 camera system). The micrographs were analyzed using Image‐Pro Plus 6.0 software. The immunoreactivity of IL‐6 in colon tissue was quantified using the H‐score, which is calculated according to the following formula: The H‐score is calculated by the following formula: H‐score = ∑(pi × *i*), where pi is the percentage of positive cells and *i* is the intensity of the specific staining. The relative intensity of specific staining was defined as follows: negative (value = 0), low positive (value = 1), positive (value = 2), and high positive (value = 3).

### 5.9 qRT‑PCR analysis

16S rRNA quantitative PCR was performed with a LightCycler 480 instrument (Roche). All reactions were performed in duplicate in one run and in duplicate PCR runs. Samples were analyzed in a 10‐μL reaction mix consisting of 6.5 μL 1× SYBR green master mix buffer (q311‐02, Vazyme), a 0.2 μM concentration of each primer, and 1 μL of genomic DNA extracted from feces. Standard curves of the full 16S rRNA PCR product of Kineothrix_alysoides and Longibaculum_muris were created using a serial 10‐fold dilution of the purified PCR product.

Quantitative PCR of colonic mRNA. Total RNA was extracted from the colon using TRIzol reagent (Invitrogen) according to the manufacturer's instructions. The following PCR conditions were used: one cycle at 95°C for 30 s, 40 cycles at 95°C for 5 s, and 60°C for 34 s. qPCR was performed using a Bio‐Rad CFX96 Touch Real‐Time PCR Detection System (Bio‐Rad Laboratories). Statistical analyses and graphing were performed using GraphPad Prism version 9. Statistical significance was set at *p* < 0.05 (two tails), unless otherwise indicated. The primer sequences are listed in Table S2.

## AUTHOR CONTRIBUTIONS

**Congcong Wang**: Supervision; formal analysis; conceptualization; methodology; software; data curation; visualization; project administration; writing—original draft; writing—review and editing. **Jinhua Lin**: Formal analysis; methodology; software; data curation; visualization; writing—review and editing. **Meng Duan**: Data curation; investigation; formal analysis; project administration; writing—review and editing. **Jialing He**: Investigation; validation; writing—review and editing; writing—original draft. **Simayi Halizere** and **Ningxin Chen**: Investigation; validation; writing—review and editing. **Xinyu Chen**, **Wei He**, and **Kenneth A Dyar**: Writing—review and editing. **Ye Jiao**: Writing—original draft; writing—review and editing. **Fei Yang** and **Shankuan Zhu**: Supervision; conceptualization; funding acquisition; project administration; resources; writing—review and editing.

## CONFLICT OF INTEREST STATEMENT

The authors declare no conflicts of interest.

## ETHICS STATEMENT

All animal experimental procedures used in this study were approved by the Zhejiang University Ethics Committee and followed the guidelines of the Zhejiang University Experimental Animal Center (approval number: ZJU20220424). All participants signed and provided informed consent forms. The human study was approved by the Ethics Committee of the School of Public Health at Zhejiang University (ZGL201905‐1).

## Supporting information

The online version contains supplementary figures and tables available.

**Figure S1:** Comparison of liver and epididymal white adipose tissue (eWAT) vacuolation areas across different dietary.
**Figure S2:** Keystone bacteria identified by quantitative polymerase chain reaction (qPCR) in feces of mice.
**Figure S3:** Amino Acid Predictors. Receiver operating characteristic (ROC) of amino acids in predicting OP mice.
**Figure S4:** Expression of mRNA (*Col6a3*, *Cyp7b1*) in colon by RNA sequencing (RNA‐seq).
**Figure S5:** Correlation heat map of fecal differential metabolites.
**Figure S6:** Distribution of microbiota in OP and OR mice at each level.
**Figure S7:** PICRUSt analysis results of predicted functional pathways in OP and OR mice.

**Table S1:** Comparison of liver and epididymal white adipose tissue (eWAT) vacuolation areas across different dietary mice groups.
**Table S2:** The sequences of primers.
**Table S3:** Characteristics of study participants in the high‐energy intake group.
**Table S4:** Differential expression of serum metabolites in OP and OR mice.
**Table S5:** Serum differential metabolites in OP and OR mouse.
**Table S6:** Enriched metabolic pathways of serum metabolites in OP and OR mice.
**Table S7:** Expression of 10 key driver genes.
**Table S8:** Enriched metabolic pathways of fecal metabolites in OP and OR mice.
**Table S9:** 16 Kyoto Encyclopedia of Genes and Genomes (KEGG) pathways overlapped between the fecal and serum metabolomes.

## Data Availability

The 16S rRNA amplicon reads of HFD‐induced C57BL/6J mice were deposited in the NCBI SRA archive under Bioproject PRJNA1048179 (https://www.ncbi.nlm.nih.gov/bioproject/PRJNA1048179). The expression profiles of mRNAs in the colon of HFD‐induced C57BL/6J mice were deposited in the Gene Expression Omnibus (GEO) database under accession number GSE249390 (https://www.ncbi.nlm.nih.gov/geo/query/acc.cgi?acc=GSE249390). Human raw sequence data reported in this study were deposited in the Genome Sequence Archive (GSA) under accession number CRA017756 (https://ngdc.cncb.ac.cn/gsa/s/LgGtbDZv), hosted by the National Genomics Data Center (China National Center for Bioinformation/Beijing Institute of Genomics, Chinese Academy of Sciences). The data and scripts used are saved in GitHub (https://github.com/candice1029/OPOR-project/tree/main/OPOR). Supplementary materials (methods, figures, tables, graphical abstract, slides, videos, Chinese translated version and update materials) may be found in the online DOI or iMeta Science http://www.imeta.science/imetaomics/.
