# Supplementary material for: Multi‐omics reveals different signatures of obesity‐prone and obesity‐resistant mice
Source: IMetaOmics. 2025 Feb 4;2(1):e59. doi: 10.1002/imo2.59 (PMC12806275; doi:10.1002/imo2.59)
Supplement: Supplementary file 1 — Figure S1: Comparison of liver and epididymal white adipose tissue (eWAT) vacuolation areas across different dietary. Figure S2: Keystone bacteria identified by quantitative polymerase chain reaction (qPCR) in feces of mice. Figure S3: Amino Acid Predictors. Receiver operating characteristic (ROC) of amino acids in predicting OP mice. Figure S4: Expression of mRNA (Col6a3, Cyp7b1) in colon by RNA sequencing (RNA‐seq). Figure S5: Correlation heat map of fecal differential metabolites. Figure S6: Distribution of microbiota in OP and OR mice at each level. Figure S7: PICRUSt analysis results of predicted functional pathways in OP and OR mice. [file IMO2-2-e59-s002.docx]

**Supporting information to**

**Multi-omics reveals different signatures of obesity-prone and obesity-resistant mice**

**Running title:** Key signatures in obesity-prone and obesity-resistant mice

Congcong Wang^1,2,4,5,6#^, Jinhua Lin^1,2#^, Meng Duan^1,2,3#^, Jialing He^1,2^, Simayi Halizere^1,2^, Ningxin Chen^1,2^, Xinyu Chen^1,2^, Ye Jiao^1^^,2^, Wei He^1,2,3^, Kenneth A Dyar^4,5^, Fei Yang^1,2,3*^, Shankuan Zhu^1,2,3*^

^1^Department of Nutrition and Food Hygiene, Children’s Hospital, Zhejiang University School of Medicine, National Clinical Research Center for Child Health, Hangzhou 310058, China.

^2^Chronic Disease Research Institute, School of Public Health, School of Medicine, Zhejiang University, Hangzhou 310058, China.

^3^Binjiang Institute of Zhejiang University, Hangzhou 310053, China.

^4^Metabolic Physiology, Institute for Diabetes and Cancer, Helmholtz Diabetes Center, Helmholtz Zentrum München, German Research Center for Environmental Health, Neuherberg 85758, Germany.

^5^German Center for Diabetes Research (DZD), Neuherberg 85764, Germany.

^6^School of Medicine and Health, Technical University of Munich, Munich 80333, Germany.

#These authors contributed equally: Congcong Wang, Jinhua Lin, Meng Duan.

*Corresponding authors: [yangfei919@zju.edu.cn](mailto:yangfei919@zju.edu.cn) (Fei Yang), [zsk@zju.edu.cn](mailto:zsk@zju.edu.cn) (Shankuan Zhu).

**Supplementary information**

**Method S1 Participant inclusion and exclusion criteria**

A total of 2,698 participants were recruited for the Lanxi cohort study. We excluded 871 participants with missing self-reported total energy intake (*n* = 188), body mass index (BMI; *n* = 74, self-reported), physical activity (*n* = 133), and fecal sample (*n* = 476) data. We also excluded 20 participants with self-reported cancer; 84 participants with extreme levels of self-reported total energy intake (females: < 500 or > 3500 kcal/day; males: < 800 or > 4200 kcal/day); 52 participants with self-reported gastritis, peptic ulcer disease, or colitis; and 61 participants with frozen-thawed fecal samples. This study focused on people who reported a high total energy intake. The ratio of total energy intake was calculated according to the recommended values for different sexes and age groups in the “Dietary Nutrient Reference Intake of Chinese Residents (2013 edition).” High energy intake was defined as an energy intake of > 110% of the estimated energy requirement.

**Figure S1 Comparison of liver and epididymal white adipose tissue (eWAT) vacuolation areas across different dietary.**

The value of *p* < 0.001 is marked with “***”. OP: obesity-prone; OR: obesity-resistant; Con: control.

**Figure S2 Keystone bacteria identified by quantitative polymerase chain reaction (qPCR) in feces of mice.** ^*^*p* < 0.05. ^**^*p* < 0.01


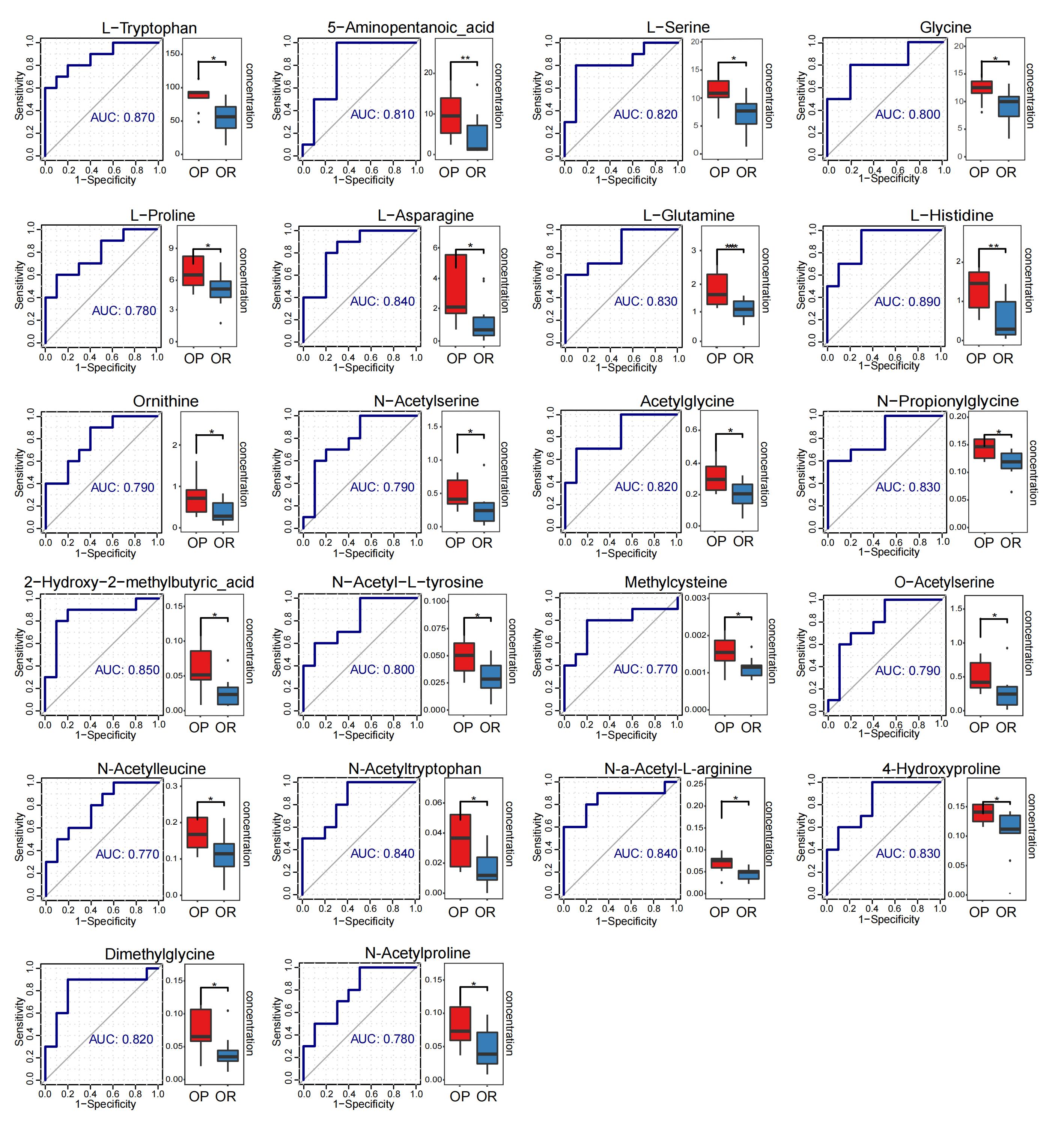
**Figure S3 Amino acid predictors.** Receiver operating characteristic (ROC) of amino acids in predicting OP mice. AUC: area under curve.

**
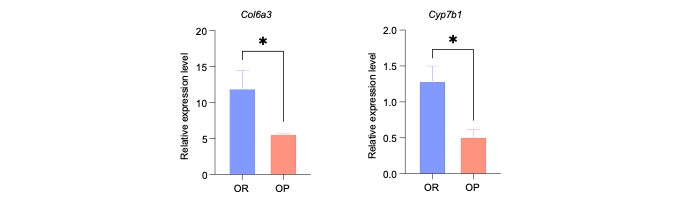
Figure S4 Expression of mRNA (*Col6a3*, *Cyp7b1*) in colon by RNA sequencing (RNA-seq).** *Col6a3* and *Cyp7b1* expression were higher in OR mice than they were in OP mice. Values represent the mean ± standard error of the mean (SEM). ^*^*p* < 0.05 (unpaired Student’s *t*-test, *n* = 3).


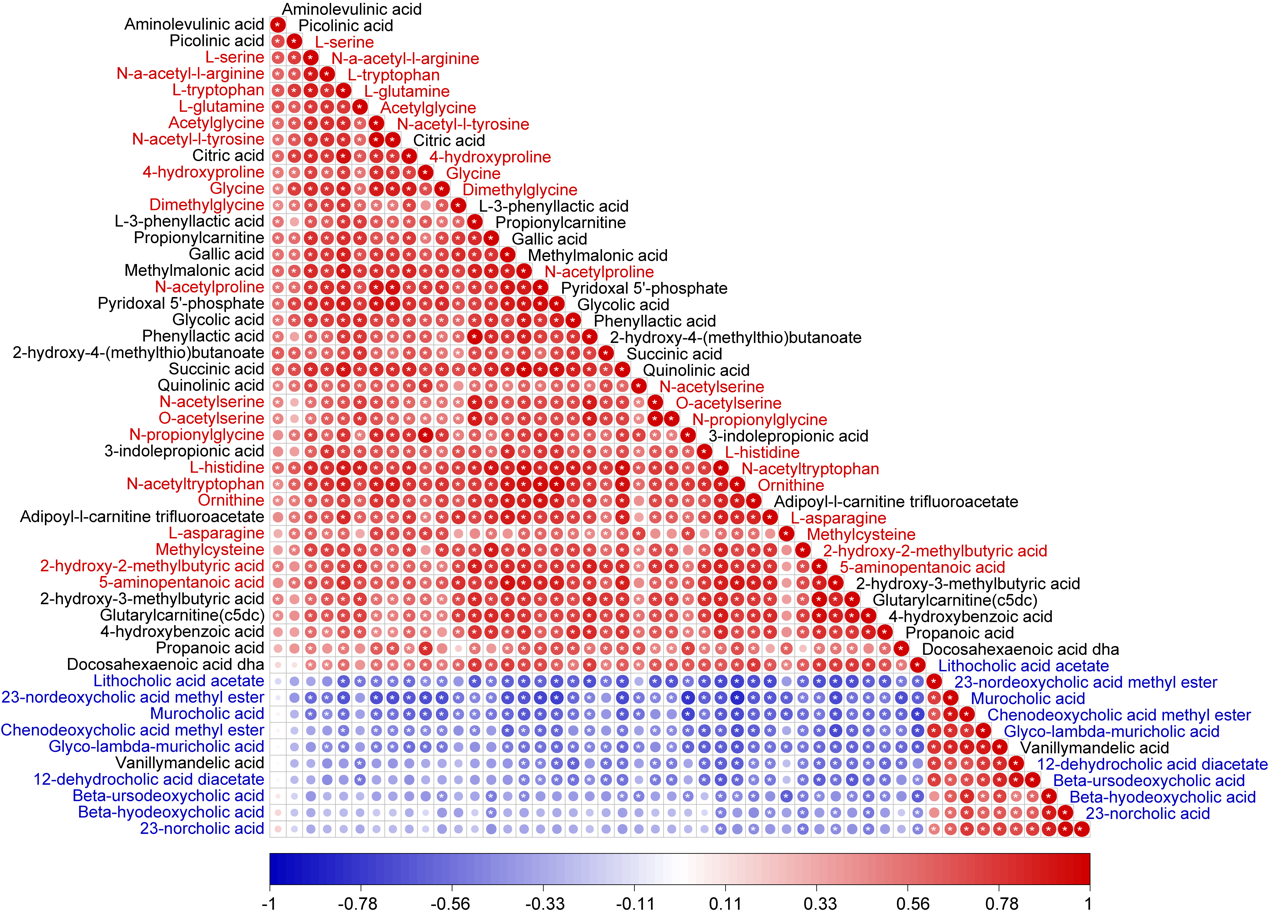


**Figure S5 Correlation heat map of fecal differential metabolites.** Red represents amino acids and blue represents bile acids. The value of *p* < 0.05 is marked with “*”.

**
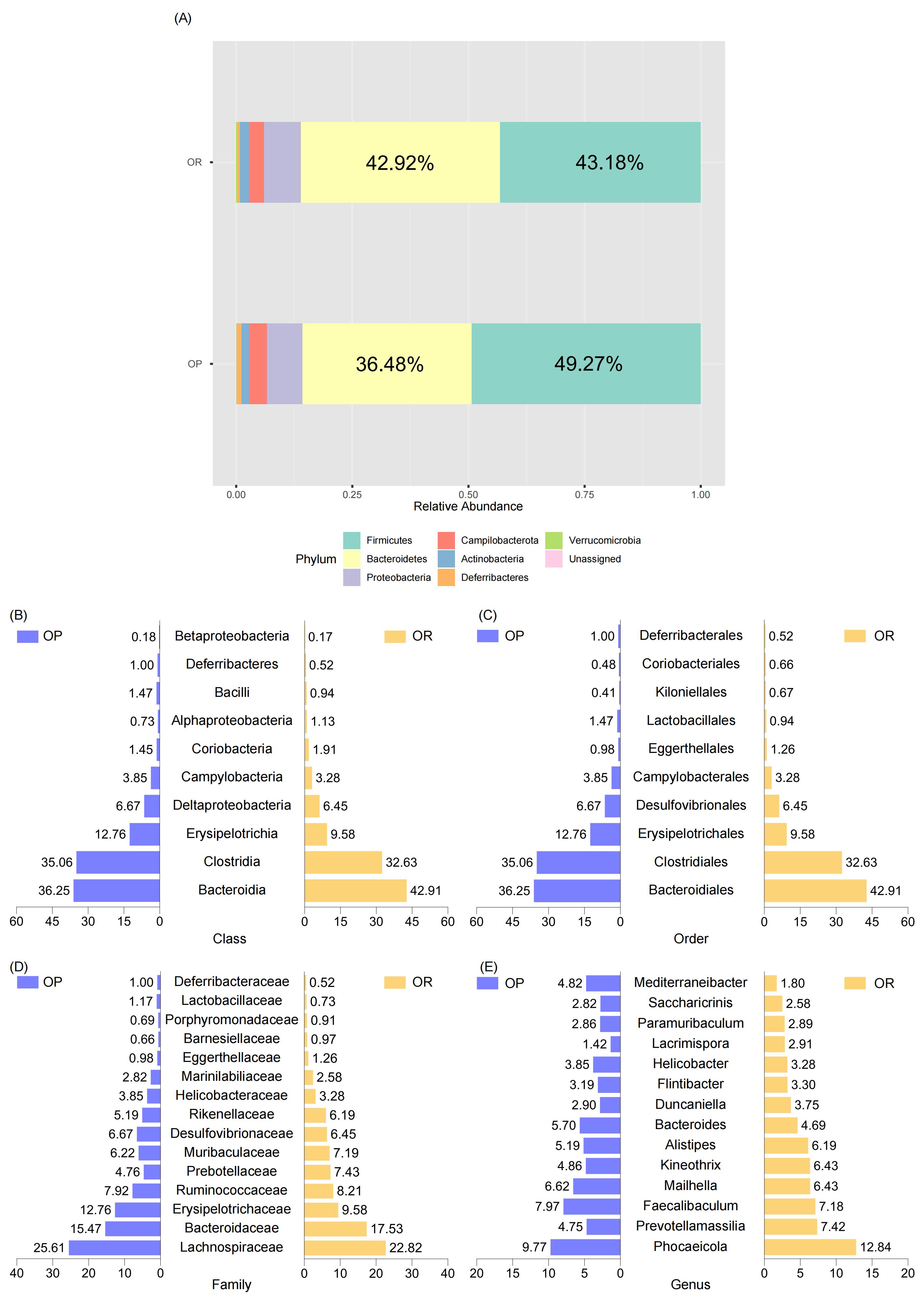
**

**Figure S6 Distribution of microbiota in OP and OR mice at each level.** (A-E) Relative abundance of the fecal microbiota at the (A) phylum, (B) class, (C) order, (D) family and (E) genus levels in OP and OR mice (*n* = 10 samples/group).

**Figure S7 PICRUSt analysis results of predicted functional pathways in OP and OR mice.** (A) Bar plot showing the relative proportions of primary and secondary bile acid biosynthesis pathways in OP and OR mice, with 95% confidence intervals. Statistically significant differences were observed, indicated by adjusted *p*-values (*p* < 0.05). (B) Box plots illustrating the sequence proportions of primary and secondary bile acid biosynthesis pathways in OP versus OR mice. OR mice show a higher predicted proportion in both pathways compared to OP mice, with asterisks denoting statistical significance (^**^*p* < 0.01).
